# Optimization of the *in vitro* Model of Cardiac Fibrosis

**DOI:** 10.21470/1678-9741-2025-0075

**Published:** 2026-05-06

**Authors:** Zeinab Neshati, Zahra Esmaeili, Farrokh Arzi

**Affiliations:** 1 Department of Biology, Faculty of Science, Ferdowsi University of Mashhad, Mashhad, Iran; 2 Institute of Biotechnology, Ferdowsi University of Mashhad, Mashhad, Iran

**Keywords:** Cardiac Fibrosis, Neonatal Rat Cardiac Fibroblasts, Angiotensin II, Ascorbic Acid, Dextran Sulfate.

## Abstract

**Introduction:**

Despite the prevalence of cardiac fibrosis, there are currently no effective treatments to reverse it. A major obstacle is the lack of reliable in vitro models. An increase in angiotensin II and collagen occurs in cardiac fibrosis. Therefore, we hypothesized that the combination of angiotensin II with ascorbic acid and dextran sulfate could induce fibrosis in neonatal rat cardiac fibroblasts (nrCFs) and be used as an in vitro model of cardiac fibrosis.

**Methods:**

nrCFs were treated with angiotensin II, ascorbic acid, and dextran sulfate. Key features of cardiac fibrosis were evaluated using Alizarin Red, Picrosirius Red, Masson's trichrome staining, quantitative reverse transcription polymerase chain reaction, and immunocytochemistry and scratch assay.

**Results:**

Although dextran sulfate increased the expression of alpha-smooth muscle actin (α-SMA), it did not increase collagen deposition and cell migration. Therefore, it seems that the combination of 500 nM angiotensin II with 100 µM ascorbic acid would be effective in induction of cardiac fibrosis in vitro, which increased (1) the expression of collagen, α-SMA, and vimentin at protein level, (2) the expression of collagen type I alpha 1 chain, collagen type III alpha 1 chain, matrix metalloproteinase 2, and transforming growth factor-beta 1 at ribonucleic acid level, and (3) cell proliferation and migration.

**Conclusion:**

In this study, 72 hour-treatment of nrCFs with 500 nM angiotensin II and 100 µM ascorbic acid was effective in the creation of an in vitro model of cardiac fibrosis. It is hoped that this model will be useful for screening antifibrotic treatments.

## INTRODUCTION

**Table:** 

Abbreviations, Acronyms & Symbols				
AA	= Ascorbic acid		FBS	= Fetal bovine serum
ACTA2	= Actin alpha 2		MMPs	= Matrix metalloproteinases
ACTB	= Actin beta		mRNA	= Messenger ribonucleic acid
Ang II	= Angiotensin II		MTT	= 3-(4,5-dimethylthiazol-2-yl)-2,5-diphenyltetrazolium bromide
AT1	= Ang II type I		nrCFs	= Neonatal rat cardiac fibroblasts
cDNA	= Complementary deoxyribonucleic acid		OD	= Optical density
Col1a1	= Collagen type I alpha 1 chain		PBS	= Phosphate buffer saline
Col3a1	= Collagen type III alpha 1 chain		RAAS	= Renin-angiotensin-aldosterone system
CTGF	= Connective tissue growth factor		RNA	= Ribonucleic acid
Dex	= Dextran sulfate		RT-PCR	= Reverse transcription polymerase chain reaction
DMEM	= Dulbecco’s modified Eagle’s medium		α-SMA	= Alpha-smooth muscle actin
DNase I	= Deoxyribonuclease I		TGF-β	= Transforming growth factor-beta
ECM	= Extracellular matrix			

Fibrotic disorders (including cardiac fibrosis, chronic kidney fibrosis, idiopathic pulmonary fibrosis, liver cirrhosis, and systemic sclerosis) and resultant organ failure account for at least onethird of deaths worldwide^[1]^. Fibrosis is one of the most devastating consequences of heart disease and has a direct role in causing heart failure, which is a major cause of mortality globally^[2]^. Cardiac fibrosis is characterized by the uncontrolled activation of cardiac fibroblasts, including excessive proliferation and differentiation, which leads to the accumulation of extracellular matrix (ECM) proteins (in particular, interstitial types I, III, and V collagens) and expansion of the cardiac interstitium^[3^,^4]^. As the major cell type in the mature myocardium, cardiac fibroblasts play an important role in maintaining ECM protein homeostasis. Activation of cardiac fibroblasts and their transition to myofibroblasts is a critical step in the development of cardiac fibrosis. Myofibroblasts play a major role in the production of ECM and in regulating ECM regeneration through the production of proteases including matrix metalloproteinases (MMPs) as well as their inhibitors^[5]^. Currently, cardiac fibrosis cannot be surgically reversed or even stopped once it has started^[3^,^4]^. Despite a relatively sophisticated understanding of the biological processes underlying fibrosis, effective treatments that specifically target cardiac fibrosis are not available. Thus far, attempts to discovery of effective antifibrotic compounds have been hampered^[6]^. A major obstacle to progress has been the lack of reliable *in vitro* models with sufficient throughput to screen effective compounds against cardiac fibrosis and to mimic the long-term trend of *in vivo* pathological conditions. As a result, there is a pressing and unmet need to develop safe and effective, reproducible, and high-throughput cardiac fibrosis models to assess the efficacy of new therapies^[5]^. *In vitro* models, despite being a simplified representation of actual diseased tissue, can be valuable because they allow for the identification of specific cellular and molecular mechanisms and drug discovery and validation in a personalised manner^[5]^. Therefore, in this study, an attempt has been made to develop an *in vitro* model able to evaluate effective therapies for cardiac fibrosis.

Cardiac fibrosis is a complex process regulated by multiple molecular pathways. Fibroblast activation involves the stimulation of the transforming growth factor-beta (TGF-β) and angiotensin II (Ang II) signaling pathways. During the progression of cardiac fibrosis, the renin-angiotensin-aldosterone system (RAAS) is persistently engaged. Ang II is a key effector molecule of the RAAS that is increased in fibrotic hearts. Ang II is a cytokine with many biological functions that, mainly by binding to its receptor AT1 (Ang II type 1), can promote the occurrence of myocardial fibrosis. There is a crosstalk between Ang II and TGF-β signaling to mediate collagen homeostasis. Ang II enhances TGF-β1 expression and activates TGF-β signaling to increase collagen production in fibroblasts^[7]^. Since in the cardiovascular system, type I collagen usually exists in the form of thick fibers with high tensile strength, the level of type I collagen in the heart is a major determinant of the proliferation of cardiac fibroblasts and is directly related to heart failure^[8]^. It is worth noting that macromolecular crowding enhances the enzymatic conversion of procollagen to collagen *in vitro*. Dextran sulfate is a sulfated polycarbohydrate polymer that, as a crowding agent, leads to a granular deposition of collagen and facilitates the formation of ECM *in vitro*^[9]^. In addition, ascorbic acid, known as vitamin C, is a crucial cofactor for a variety of enzymes involved in collagen synthesis and maturation, including prolyl-3-hydroxylase, prolyl-4-hydroxylase, and lysyl hydroxylase^[10]^. Therefore, in this study, we decided to create a stable *in vitro* model using Ang II, ascorbic acid, and dextran sulfate to investigate cardiac fibrosis.

## METHODS

### Isolation and Culture of Neonatal Rat Cardiac Fibroblasts

This study followed animal procedures institutional and international ethical guidelines with adherence to the ARRIVE guidelines, as required by the Brazilian Journal of Cardiovascular Surgery, and was approved by the Animal Ethics Committee of the Ferdowsi University of Mashhad (Approval ID: IR.UM.REC.1400.130). Neonatal rat cardiac fibroblasts (nrCFs) were isolated from neonatal (1 - 3 days old) Wistar rats. Briefly, left ventricles (n = 12) were collected, cut into pieces, and digested with 450 units/ml of collagenase 1 (Sigma-Aldrich, Germany) and 18.75 Kunitz units/ml deoxyribonuclease I (DNase I) type IV (Sigma-Aldrich, Germany). After centrifugation and resuspension in Ham’s F10 medium (Sigma-Aldrich, Germany) supplemented with 10% fetal bovine serum (FBS) (Gibco™, Thermo Fisher Scientific, United States of America) and 10% horse serum (Sigma-Aldrich, Germany), cells were plated in a cell culture dish for 75 minutes to separate and purify nrCFs using the different speed adherence method. Then, the cells were cultured in low-glucose Dulbecco’s modified Eagle’s medium (DMEM) (Gibco™, Thermo Fisher Scientific, United States of America) supplemented with 10% FBS (Gibco™, Thermo Fisher Scientific, United States of America) at 37°C in 5% CO2. nrCFs at passage 2 were used for subsequent experiments. Each experiment was performed three times, and three separate cell wells were used for each time.

### Cell Cytotoxicity Assay

To evaluate the cell proliferation and the cytotoxicity effect of Ang II, ascorbic acid, and dextran sulfate on nrCFs, cells were cultured in a 96-well plate (104 cells/well) for 24 hours to reach about 80% confluency. After serum-starvation for 48 hours, nrCFs were treated with Ang II (Sigma-Aldrich, Germany), ascorbic acid (Titrachem, Iran), and dextran sulfate (Bio Basic, Canada) alone and in combination with each other for 72 hours. 20 µl 3-(4,5-dimethylthiazol-2-yl)-2,5-diphenyltetrazolium bromide (MTT) (ATOCEL, Hungary) working solution (5 mg/ml in phosphate buffer saline [PBS], [Zist Mavad Pharmed, Iran]) was added to each well and incubated at 37°C for four hours. To dissolve formazan crystals that were formed by viable cells, the medium was replaced with 200 µL dimethyl sulfoxide (SCHARLAU, Spain). Then the optical density (OD) was measured at 545 nm using an enzyme-linked immunosorbent assay reader (Awareness Technology, Stat Fax® 2100, United States of America). Cells cultured in serum-free DMEM (Gibco™, Thermo Fisher Scientific, United States of America) were used as negative control.

### Cell Treatments

Ang II (Sigma-Aldrich, Germany) and ascorbic acid (Titrachem, Iran) were dissolved in serum-free DMEM (Gibco™, Thermo Fisher Scientific, United States of America) and were stored at -80 and -20°C, respectively. Dextran sulfate (Bio Basic, Canada) was dissolved in deionized water and stored at 4°C. Before treatment, nrCFs were starved in serum-free medium for 48 hours. Then, the cells were treated with different concentrations of Ang II (100, 500, and 1000 nM), ascorbic acid (100, 280, and 500 µM) and dextran sulfate (100 µg/ml) separately and in combination for 72 hours. The selected concentrations of Ang II and ascorbic acid were based on the cell viability and morphology microscopic assays.

### Masson’s Trichrome Staining

For detection of collagen fibers, nrCFs were cultured in a 96-well plate (104 cells/well) for 24 hours before treatment. After serum starvation for 48 hours, the nrCFs were treated with Ang II, ascorbic acid, and dextran sulfate for 72 hours. Then cells were fixed in 4% formaldehyde solution (Merck, Germany) for 30 minutes and re-fixed in Bouin's solution (picric acid [Merck, Germany], formaldehyde solution [Merck, Germany], acetic acid [Merck, Germany]) for one hour to improve staining quality. Then, nrCFs were embedded in tap water for 10 minutes and stained with Weigert's iron hematoxylin working solution for 40 minutes. After embedding in tap water for 10 minutes, nrCFs were stained with Biebrich scarlet-acid fuchsin solution for 60 minutes. After washing the cells with tap water, they were stained with phosphomolybdic-phosphotungstic acid solution (Merck, Germany) for three hours, followed by aniline blue staining (Merck, Germany) for 12 hours. Then cells were washed three times in 1% acetic acid solution (Merck, Germany) and were kept in PBS (Zist Mavad Pharmed, Iran). The collagen fibers were stained blue, which were examined under an inverted microscope (Zeiss Axiovert S100 HP, Germany).

### Picrosirius Red Staining

To evaluate the relative levels of insoluble collagen accumulation in nrCFs, cells were cultured as described previously. After treatment period, cells were rinsed three times with ice-cold PBS and were fixed with Bouin’s solution for one hour at room temperature. Cells were rinsed several times with tap water until total removal of the yellow color of Bouin. Overnight air-dried cells were embedded in Picrosirius red dye solution for three hours at room temperature, washed four times with 0.01 M HCl, and photographed by a digital camera mounted on inverted microscope. Subsequently, for spectrophotometric analysis, bound Picrosirius red (Merck, Germany) was eluted in 0.1 M NaOH (Merck, Germany) by mixing on an orbital shaker for 30 minutes. Supernatants were placed on another 96-well plate, and the OD was measured at 545 nm.

### Ribonucleic Acid Extraction and Real-Time Reverse Transcription Polymerase Chain Reaction

The total ribonucleic acid (RNA) was extracted from nrCFs using TRIzol™ reagent according to manufacturer instruction (Total RNA Isolation Kit, Deazist, Iran). The isolated RNA (1000 ng) was treated with DNase I (Thermo Fisher Scientific, United States of America) in 11 µl total reaction volume, and afterwards 8 µl of DNase I-treated RNA were reverse transcribed into complementary deoxyribonucleic acid (cDNA) using M-MLV reverse transcriptase based on manufacturer instruction (cDNA Synthesis Kit, Parstous, Iran). Real-time PCR was performed using an SYBR® Green Real Time PCR kit (Parstous, Iran) to detect the level of messenger RNA (mRNA) expression of MMP2, collagen type I alpha 1 chain (Col1a1) and collagen type III alpha 1 chain (Col3a1), actin alpha 2 (ACTA2) smooth muscle, and TGF-β1 genes. The level of mRNA expression was normalized to that of the house-keeping gene actin beta (or ACTB) as its expression was not changing during the experimental procedure in different groups. The 2-∆∆Ct method was used for data analysis. The primer sequences (Metabion, Germany) have been presented in Table 1. All primers for real-time PCR were designed to have annealing temperatures of ∼60°C and to give amplicons of ∼200 base pairs in length and to be positioned on different exons (exon-spanning primers) to prevent genomic deoxyribonucleic acid amplification. The efficiency of the primers was determined using LinRegPCR software.

**Table 1:** Sequence of the primers.

Primer	Sequence	
ACTB	Forward	5'- AAGATGACCCAGATCATGT -3'
	Reverse	5'- AGGTCCAGACGCAGGATG -3'
Col1a1	Forward	5'- GGCAAGAACGGAGATGAT -3'
	Reverse	5'- CACCATCCAAACCACTGA -3'
Col3a1	Forward	5'- AAGGCTGAAGGAAATAGCA -3'
	Reverse	5'- CAGGACCACCAATGTCATA -3'
ACTA2	Forward	5'- CTGCTGCTTCCTCTTCTT -3'
	Reverse	5'- TATAGGTGGTTTCGTGGATG -3'
MMP2	Forward	5'- TGACCTTGACCAGAACAC -3'
	Reverse	5'- GCATCATCCACTGTCTCA -3'
TGF-β1	Forward	5'- GGATACCAACTACTGCTTCA -3'
	Reverse	5'- GTCCAGGCTCCAAATGTA -3'

ACTA2=actin alpha 2; ACTB=actin beta; Col1a1=collagen type I alpha 1 chain; Col3a1=collagen type III alpha 1 chain; MMP2=matrix metalloproteinase 2; TGF-β=transforming growth factor-beta

### Immunocytology

To check the expression of alpha-smooth muscle actin (α-SMA) and vimentin proteins, nrCFs were fixed with 4% formaldehyde (Merck, Germany) for 30 minutes at room temperature, permeabilized with PBS (Zist Mavad Pharmed, Iran) containing 0.1% Triton™ X-100 (Betacell, Iran), and blocked with PBS containing 5% bovine serum albumin (Merck, Germany). Next, the cells were incubated with primary antibodies against α-SMA (mouse, Zytomed Systems) or vimentin (1:400, mouse, Sigma, Germany) 75 minutes at room temperature and overnight at 4°C, respectively. This was followed by two-hour incubation at room temperature with Alexa Fluor 568 Donkey anti-mouse (1:400, Life Technologies, United States of America) secondary antibody. 4′,6-Diamidino-2-phenylindole + Antifade solution (DENAzist, Iran) was used to visualize the nuclei. The cells were observed under a Nikon ECLIPSE Ti-S microscope (Nikon, Japan). The intensity of the signals and the number of positive cells were measured by Image J software in five randomly chosen fields.

### Cell Migration Assay (Scratch Assay)

Cells were incubated in a serum-free medium for 48 hours. A single scratch was created in the center of the cell monolayers by gently scraping the attached cells with a sterile 1-mL micropipette tip. Then cells were immediately placed in serum-free media containing Ang II, ascorbic acid, and dextran sulfate for 72 hours. The cells in control group were treated with serum-free media for 72 hours. The width of the scratched area was assessed by comparing micrographs at time 0 and 72 hours by ImageJ software. The percentage of wound closure was calculated using Equation 1:


Wound Closure%=[(initial wound area - wound area after 72 hours)/initial wound
area]×100

### Statistical Analysis

All data are expressed as the means ± standard deviation. Comparisons between groups were performed using either an unpaired Student's *t*-test or one-way analysis of variance with GraphPad Prism 9.0 software. Differences at *P* values ≤ 0.05 were considered significant.

## RESULTS

### Cell Morphology and Viability

**Fig. 1:**
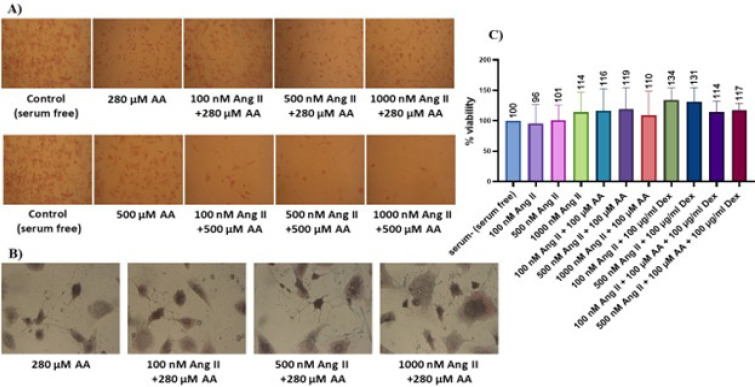
A and B) Change in durability and morphology of neonatal rat cardiac fibroblasts (nrCFs) during treatment with high concentrations of ascorbic acid (AA). C) Evaluation of the cytotoxic effects of angiotensin II (Ang II) (alone or in combination with AA and dextran sulfate [Dex]).

Different concentrations of Ang II (100, 500, and 1000 nM), ascorbic acid (100, 280, and 500 µM), and dextran sulfate (100 µg/ml) were used separately and in combination to create cardiac fibrosis. Treatment of nrCFs with concentrations of 280 and 500 µM of ascorbic acid, either individually or in combination with Ang II, decreased the cell durability (Figure 1A) and caused malformation in the cells (Figure 1B). Therefore, these concentrations were excluded from the treatments. MTT assay was performed after treatment with lower concentrations of ascorbic acid. The results did not show significant toxicity and cell death (Figure 1C).

### Masson’s Trichrome Staining

**Fig. 2:**
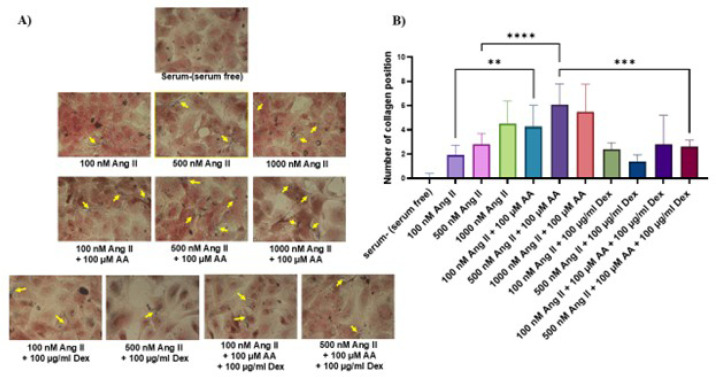
Collagen accumulation in neonatal rat cardiac fibroblasts cultures stimulated by angiotensin II (Ang II), ascorbic acid (AA), and dextran sulfate (Dex). A) Addition of AA and increment in the concentration of Ang II increased the number of collagen fibers, while Dex treatment reduced collagen synthesis. The collagen fibers were stained blue (yellow arrows). B) Quantification of collagen fibers. Data are expressed as the mean ± standard deviation (three separate experiments with three replicates in each). **P < 0.01; ***P < 0.001; and ****P < 0.0001.

The changes in collagen expression were detected by Masson’s trichrome staining. Addition of ascorbic acid and increment in the concentration of Ang II increased the number of collagen fibers, while dextran sulfate had negative effects on collagen synthesis. Treatment of cells with 100 µM of ascorbic acid in combination with 1000 nM Ang II resulted in death and malformation of the cells (Figure 2).

### Cell Migration

**Fig. 3:**
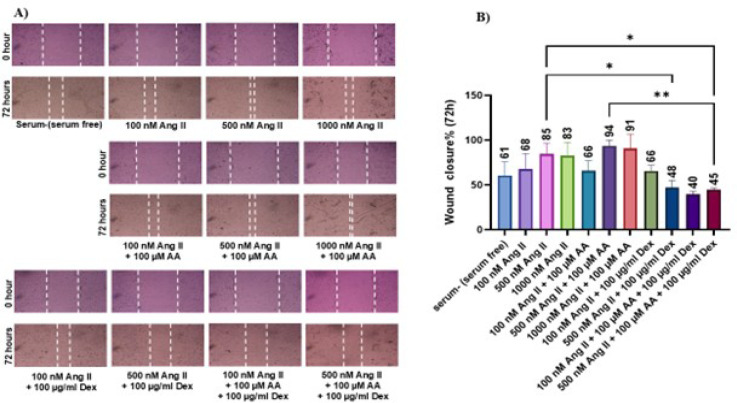
Representative images of scratch closure. A) Represents the wound area at 0 and 72 hours after treatment. (B) The percentage of wound closure after 72 hours. The significance of the difference between the groups has been evaluated in such a way that the treatment effect of one agent, namely ascorbic acid (AA) or dextran sulfate (Dex), on cell migration be measured. Data are expressed as mean ± standard deviation (three separate experiments with three replicates in each). *P < 0.05; **P < 0.005. Ang II=angiotensin II.

To investigate the effect of Ang II, ascorbic acid, and dextran sulfate on the migration capacity of nrCFs, scratch assay was performed. Increment in the concentration of Ang II and addition of ascorbic acid did not result in a significant increase in cell migration ability, but dextran sulfate had significant inhibitory effect on migration (Figure 3).

### Picrosirius Red Staining

**Fig. 4:**
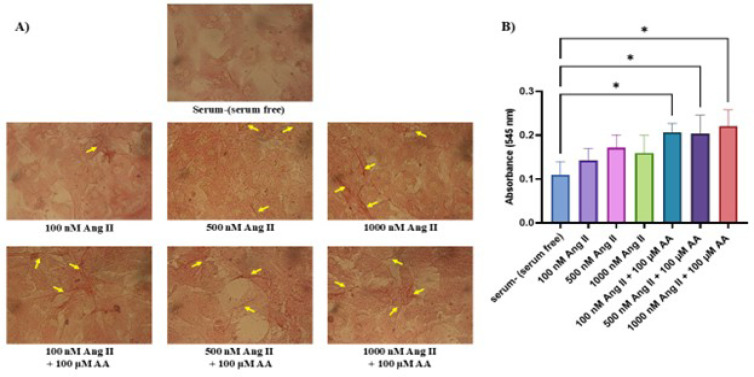
Collagen accumulation in neonatal rat cardiac fibroblasts cultures treated with angiotensin II (Ang II) and ascorbic acid (AA). A) Yellow arrows indicate collagen fibers in red. B) Quantification of the amount of absorbed dye. *P < 0.05.

In Picrosirius Red staining, collagen fibers are stained in red. The number of collagen fibers was increased in higher concentrations of Ang II and by ascorbic acid treatment (Figure 4A). Absorption results also showed a significant increase in Ang II and ascorbic acid combined treatments compared to the control group (Figure 4B).

### Expression of Alpha-Smooth Muscle Actin at Protein Level

**Fig. 5:**
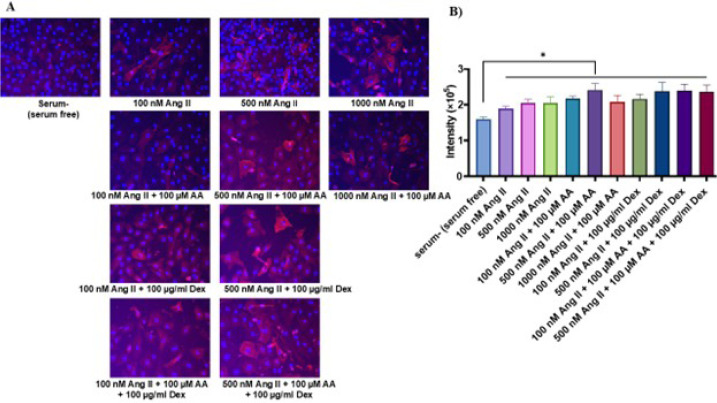
The expression of alpha-smooth muscle actin (α-SMA) protein. A) Immunostaining of α-SMA. (B): Quantification of α-SMA intensity expression in positive cells. Data are expressed as mean ± standard deviation (three separate experiments with three replicates in each). *P < 0.05. AA=ascorbic acid; Ang II=angiotensin II; Dex=dextran sulfate.

All treatments showed a significant increase in α-SMA expression compared to the control. The highest increase (1.5-fold) in the expression was observed in the combined treatment of 500 nM Ang II and 100 µM ascorbic acid (Figure 5A and 5B).

### Expression of Vimentin at Protein Level

**Fig. 6:**
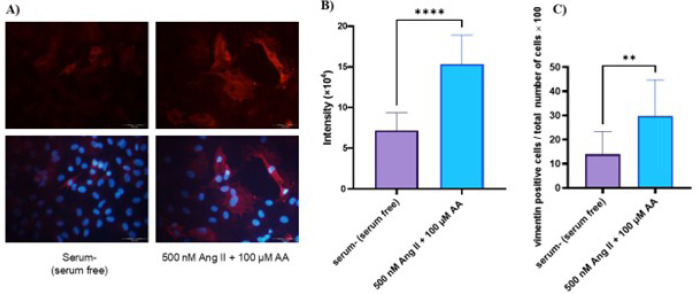
The expression of vimentin at protein level. A) Immunostaining of vimentin. B) Quantification of vimentin signal intensity in positive cells. C) The percentage of vimentin positive cells (red) out of total number of nuclei (blue). Data are expressed as mean ± standard deviation (three separate experiments with three replicates in each). **P < 0.01; ****P < 0.0001. AA=ascorbic acid; Ang II=angiotensin II.

The expression of vimentin was assessed in 500 nM Ang II and 100 µM ascorbic acid treatment as these treated cells showed the highest amount of α-SMA protein expression. The results showed around a 2-fold-increase in vimentin expression after treatment with 500 nM Ang II and 100 µM ascorbic acid (Figure 6A and 6B). The number of positive cells was also increased by fibrosis induction (Figure 6C).

### Expression of Fibrotic Markers at Ribonucleic Acid Level

**Fig. 7:**
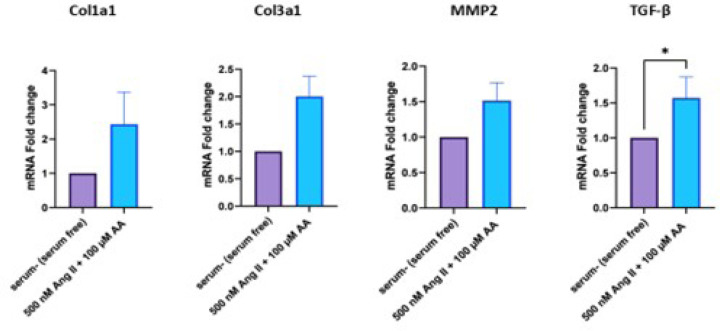
Messenger ribonucleic acid (mRNA) levels of collagen type I alpha 1 chain (Col1a1), collagen type III alpha 1 chain (Col3a1), matrix metalloproteinase 2 (MMP2), and transforming growth factor-beta (TGF-β) in neonatal rat cardiac fibroblasts (nrCFs) were evaluated by real-time reverse transcription polymerase chain reaction between angiotensin II (Ang II) and ascorbic acid (AA)-treated nrCFs and control group. Data are expressed as mean ± standard deviation (three separate experiments with three replicates in each). *P < 0.05.

To investigate the expression of Col1a1, Col3a1, TGF-β1, and MMP2 genes at the RNA level after 500 nM Ang II and 100 µM ascorbic acid treatment, real-time RT-PCR was conducted. Results showed 2.4-, 2-, 1.-5, and 1.57-fold increase in the expression of Col1a1, Col3a1, MMP2, and TGF-β1 genes, respectively (Figure 7).

## DISCUSSION

The molecular basis of fibrosis in the heart involves excessive deposition of ECM components and increased expression of profibrotic genes such as proline-rich collagens, MMPs, connective tissue growth factor (CTGF), etc. Collagens are likely to be the main determinants of cardiac fibrosis, along with other molecules such as fibronectin, elastin, fibrillin, and CTGF. Cardiac fibroblasts, the most predominant cell type in the myocardium, are known to be the main source of synthesis of these molecules^[11^,^12]^. Although, cardiac fibrosis has critical importance in cardiovascular diseases, our limited understanding of the cardiac fibroblasts impedes the development of potential therapies that effectively target this cell type and its pathological contribution to disease progression^[13]^. Since cardiac fibroblasts play a crucial role in the progression of fibrosis and regeneration, they can be utilized to study various aspects of cardiac fibrosis and to create a model that closely mimics the disease. The primary symptom of fibrosis is the differentiation of fibroblasts into myofibroblasts, which are the primary cellular effectors of fibrosis. Myofibroblasts are the phenotypic cell variants derived from quiescent fibroblasts. The most commonly used standard marker for identifying cardiac myofibroblasts is α-SMA protein, which is poorly expressed in cardiac fibroblasts^[14]^. Myofibroblasts highly express vimentin and have increased proliferative and migratory properties, as well as enhanced capacity for synthesizing ECM components, such as collagen. Myofibroblasts also secrete proteolytic enzymes, including MMPs (specifically, MMP2 and MMP9), which are responsible for ECM degradation. The activation of fibroblasts into myofibroblasts is regulated by various soluble factors including cytokines, growth factors, and oxidative stress products. Among the molecules involved, TGF-β1 and Ang II play a pivotal role in triggering and sustaining fibrogenesis. Several heart failure models have shown that elevated levels of Ang II and TGF-β1 are associated with myocardial fibrosis. To simulate cardiac fibrosis *in vitro*, cultured neonatal cardiac fibroblasts can be treated with these growth factors^[15]^. Numerous studies have demonstrated that TGF-β1 treatment of cardiac fibroblasts *in vitro* induces the differentiation of these cells into myofibroblasts and increases ECM protein synthesis. Ang II, on the other hand, increases the synthesis of ECM proteins such as fibronectin and the secretion of multiple cytokines, including TGF-β1. TGF-β acts as a downstream mediator of Ang II, enhancing its pathological role in inducting cardiac fibrosis. Ang II can induce fibrosis independently and in synergy with TGF-β, promoting collagen I expression directly and independently of TGF-β1 through the AT1-ERK/p38 MAPK cross-talk pathway^[16^,^17]^. Additionally, Ang II increases the expression of fibrotic genes primarily TGF-β1, MMP2, MMP9, and ACTA2, at both the RNA and protein levels^[18^,^19]^. In addition to Ang II and TGF-β1, vitamin C (ascorbic acid) also plays an important role in fibroblast differentiation. It has been shown that vitamin C enhances the expression of α-SMA and ACTA2 in human dermal fibroblasts^[20]^. In addition, researchers have shown that without vitamin C, collagen cannot be synthesized properly^[10]^. Ascorbic acid is a water-soluble vitamin which acts as a cofactor for essential enzymes in the biosynthesis of collagen, especially types I and type III. Briefly, the synthesis of collagen involves a number of post-translational modifications, notably proline and lysine hydroxylation, lysine glycosylation, and N- and C-terminal propeptide cleavage. Ascorbic acid regulates the biosynthesis and posttranslational modifications of collagen by maintaining the activity of the enzyme prolyl hydroxylase^[10^,^21]^. This enzyme plays a central role in the formation of the triple helix structure by hydroxylation of proline and lysine residues. Following additional modifications such as glycosylation of lysine, the triple helical molecule becomes the procollagen molecule, flanked by globular N- and C-terminal propeptides and is packaged and secreted into the extracellular space. Collagen deposition into a stable matrix depends on the enzymatic extracellular conversion of procollagen to collagen^[22]^. Dextran sulfate is a sulfated poly carbohydrate polymer and induces processing of procollagen to mature collagen. Dextran sulfate increases the interaction of the procollagen with C and N proteinases and accelerate enzymatic removal of C- and N-peptides of cell-secreted procollagen. Trimming off the propeptides by peptidases leads to the creation of a mature collagen with triple helix form that self-assembles into fibrils followed by formation of covalent cross-links initiated by lysyl oxidase. Dextran sulfate also accelerates the collagen and lysyl oxidase interaction^[23]^. In addition, ascorbic acid increases the production of metalloproteinase inhibitors, avoiding the degradation of existing collagen^[24]^.

In this research, an attempt was made to create an optimal *in vitro* model of cardiac fibrosis using the treatment of nrCFs with Ang II, ascorbic acid, and dextran sulfate.

To verify whether the combination of Ang II, ascorbic acid, and dextran sulfate induces collagen synthesis in nrCFs, we treated the cells with Ang II (100 nM, 500 nM, 1000 nM), ascorbic acid (100 µM), and dextran sulfate (100 µg/ml) for 72 hours, and performed the subsequent experiments. In this study, Picrosirius Red and Masson's trichrome staining were used to investigate and compare collagen expression changes. In evaluation of collagen expression using Masson's trichrome and Picrosirius Red staining, collagen fibers were observed in all treatments. In the combined treatment with a concentration of 500 nM Ang II and 100 µM ascorbic acid, as well as in the combined treatment with a concentration of 1000 nM Ang II and 100 µM ascorbic acid, the number of collagen fibers was higher than in other groups. The results indicate the effectiveness of Ang II and ascorbic acid treatment in induction of collagen synthesis by myofibroblasts. According to the results of most articles, the use of dextran sulfate was expected to increase the conversion of procollagen to collagen and, as a result, increase in collagen deposition. But the results of the present study showed that the addition of dextran sulfate reduced the increasing effect of Ang II and ascorbic acid in collagen expression. The higher concentrations of the 500 nM AngII resulted in abnormalities in cell morphology. Therefore, 500 nM Ang II and 100 µM ascorbic acid were selected for the treatment of cells to investigate gene expression changes (Col1a1, Col3a1, TGF-β1, and MMP2) using quantitative real-time PCR. Our results showed that the expression of Col1a1, Col3a1, MMP2, and TGF-β1 genes was increased by 2.4, 2, 1.5, and 1.57 times, respectively, at the RNA level.

We also investigated the expression of α-SMA and vimentin proteins using immunocytochemistry. The intensity of vimentin expression and the percentage of vimentin positive cells was around 2 times higher than the control group. α-SMA protein expression also showed a significant increase in all treatments compared to the control. These results indicate the relative effectiveness of the 500 nM Ang II and 100 µM ascorbic acid treatment in transforming nrCFs into myofibroblast and induction of fibrosis.

Myofibroblasts are not static and have increased migratory properties that allow them to migrate to the damaged myocardium in response to chemokines released at the site of injury^[25^,^26]^. Therefore, in this study, the scratch assay was used to investigate the differentiation of fibroblasts into myofibroblasts and the migration rate of these cells. Although an increase in cell migration was observed after Ang II treatment, this increase was not significant. Contrary to our expectation, dextran sulfate decreased the migration rate.

### Limitations

nrCFs are cells that are directly harvested from the heart tissue and retain relatively similar qualities to their *in vivo* phenotype. However, the usage of nrCFs is restricted by some important limitations. These cells have a limited lifespan and a low proliferation rate. Moreover, cell responses and behavior of nrCFs may be altered at different passages, which creates translational gaps to human pathophysiology^[27^,^28]^.

## CONCLUSION

Cardiac fibrosis is a complex, multistep cellular and molecular process that occurs from weeks to months *in vivo* and has, therefore, proven difficult to recapitulate *in vitro*. However, cardiac fibrosis is accompanied by other cardiac complications *in vivo* (inflammation and cardiac hypertrophy), which make it difficult to understand its contribution to heart failure separately. Therefore, in this study, we decided to create an *in vitro* model to investigate cardiac fibrosis independent of other cardiac complications. In this regard, Ang II was used for initial induction of the fibrosis in nrCFs, followed by the addition of ascorbic acid and dextran sulfate to investigate whether they can enhance the induction of fibrosis. Considering that, the addition of ascorbic acid simultaneously with Ang II had a great effect on improving the expression of TGF-β1, MMP2, collagen, vimentin, α-SMA, and cell migration, but the addition of dextran sulfate, despite increasing the expression of α-SMA, did not increase the collagen deposition, proliferation, and cell migration. It appears that the combined treatment of 500 nM Ang II and 100 µM ascorbic acid is effective in differentiating nrCFs into myofibroblasts and creating an optimal *in vitro* model of cardiac fibrillation. This proposed model has potential translational applications and can be applied in preclinical testing or screening of antifibrotic therapies. First, it will enable the study and discovery of new treatments for cardiac fibrosis, and second, it will enable screening for adverse drug reactions on the heart. It is hoped that using this model in preclinical development will lead to drugs with better efficacy and safety parameters *in vivo* and in the clinic.

## Data Availability

The authors declare that the raw data supporting the findings of this study will be available upon reasonable request to the authors, but the analyzed data are available within the article.

## Artificial Intelligence Usage

The authors declare that no artificial intelligence tool was used in the preparation of this article.
